# Combining an *in silico* proarrhythmic risk assay with a tPKPD model to predict QTc interval prolongation in the anesthetized guinea pig assay

**DOI:** 10.1016/j.taap.2020.114883

**Published:** 2020-03-01

**Authors:** Pierre Morissette, Sebastian Polak, Anne Chain, Jin Zhai, John P. Imredy, Mary Jo Wildey, Jeffrey Travis, Kevin Fitzgerald, Patrick Fanelli, Elisa Passini, Blanca Rodriguez, Frederick Sannajust, Christopher Regan

**Affiliations:** a Safety Assessment & Laboratory Animal Resources (SALAR), Merck & Co., Inc., West Point, PA, USA; b Certara UK Limited, Simcyp Division, Sheffield, UK; c Pharmacokinetics, Pharmacodynamics and Drug Metabolism (PPDM), Merck & Co., Inc., Rahway, NJ, USA; d Pharmacology, Screening and Informatics, Merck & Co., Kenilworth, NJ, USA; e Computational Cardiovascular Science Group, Department of Computer Science, BHF Centre of Research Excellence, University of Oxford, Oxford, UK; f Jagiellonian University Medical College, Faculty of Pharmacy, Krakow, Poland

**Keywords:** Anesthetized Cardiovascular Guinea Pig, *In Silico* modeling, QT corrected interval, Torsade de Pointes, Translational PKPD modeling, Safety pharmacology, AP, Action Potential, APD_90_, Action Potential Duration at 90% repolarization, BP, Blood Pressure, CVGP, Cardiovascular Anesthetized Guinea Pig, CTD_90_, Ca^2+^-Transient Duration at 90% repolarization, ECG, Electrocardiogram, EMw, Electromechanical window, FIH, First in Human, FLIPR, Fluorescent Imaging Plate Reader, FN, False Negative, FP, False Positive, GP, Guinea Pig, hERG, Human Ether-a-go-go Related Gene, HR, Heart Rate, HTS, High Throughput Screening, ICH, The International Council for Harmonization of Technical Requirements for Pharmaceuticals for Human Use, I_Kr_, rapidly-activating delayed rectifier potassium current, LOO, Leave-One-Out, LVP, Left Ventricular Pressure, M5P, M5 trees automated pruning model, NCEs, New Chemical Entities, ORd, O'Hara Rudy model, PNV, Predictive Negative Value, PPV, Positive Predictive Value, PX, PatchXpress®, QTc, Heart Rate corrected QT Interval, QTcVdW, Van de Water's Heart Rate QT-corrected Interval, TdP, Torsade de Pointes, tPKPD, thranslational pharmacokinetic/pharmacodynamic, TN, True Negative, TP, True Positive

## Abstract

Human-based *in silico* models are emerging as important tools to study the effects of integrating inward and outward ion channel currents to predict clinical proarrhythmic risk. The aims of this study were 2-fold: 1) Evaluate the capacity of an *in silico* model to predict QTc interval prolongation in the *in vivo* anesthetized cardiovascular guinea pig (CVGP) assay for new chemical entities (NCEs) and; 2) Determine if a translational pharmacokinetic/pharmacodynamic (tPKPD) model can improve the predictive capacity. *In silico* simulations for NCEs were performed using a population of human ventricular action potential (AP) models. PatchXpress® (PX) or high throughput screening (HTS) ion channel data from respectively *n* = 73 and *n* = 51 NCEs were used as inputs for the *in silico* population. These NCEs were also tested in the CVGP (n = 73). An M5 pruned decision tree-based regression tPKPD model was used to evaluate the concentration at which an NCE is liable to prolong the QTc interval in the CVGP. *In silico* results successfully predicted the QTc interval prolongation outcome observed in the CVGP with an accuracy/specificity of 85%/73% and 75%/77%, when using PX and HTS ion channel data, respectively. Considering the tPKPD predicted concentration resulting in QTc prolongation (EC_5%_) increased accuracy/specificity to 97%/95% using PX and 88%/97% when using HTS. Our results support that human-based *in silico* simulations in combination with tPKPD modeling can provide correlative results with a commonly used early *in vivo* safety assay, suggesting a path toward more rapid NCE assessment with reduced resources, cycle time, and animal use.

## Highlights

• Cardiac electrophysiological *in silico* model predicts QTc interval prolongation in the guinea pig.
• PKPD model predicts relevant QTc interval prolongation concentration in guinea pig.
• Combining the models improves the accuracy of predicting guinea pig QTc effects.
• Combining models accelerates assessment of QTc with lower resources and animal use.

## 1 Introduction

Drug-induced cardiac proarrhythmic risk, including polymorphic ventricular tachycardic arrhythmias and, in particular, Torsade de Pointes (TdP), represents a life-threatening adverse effect. They have not only led to the withdrawal of marketed products (Heist and Ruskin, 2010), but the risk of such events continues to be a significant reason for drug development attrition. This resulted in the creation of two important documents by the International Conference on Harmonization of Technical Requirements for Registration of Pharmaceuticals for Human Use (ICH): 1) the preclinical ICH-S7B guideline describing the preclinical evaluation of cardiac proarrhythmic risk and 2) the E14 guideline discussing the evaluation of proarrhythmic risk in humans. The S7B guideline recommended the evaluation of new drug candidates for effects on I_Kr_ or Human Ether-a-go-go Relatated Gene (hERG) *in vitro* and on the QTc interval *in vivo* as primary preclinical studies to evaluate their potential for drug-induced QTc interval prolongation and arrhythmogenesis. Although these ICH guidelines were in response to a critical health concern, their reliance solely on hERG inhibition and/or QTc interval prolongation may have limited the advancement of potentially safe and effective drugs that caused QTc interval prolongation but with no or acceptable arrhythmogenic potential to market (Salvi et al., 2010).

It is widely accepted that the major cellular mechanism enabling TdP is the blockade of the rapidly activating delayed rectifier potassium current (I_Kr_), encoded by *hERG* (or *KCNH2*). However, it has been well documented that: 1) hERG inhibition on its own does not always translate to the occurrence of TdP, 2) action potential duration (APD) prolongation *in vitro* is not always associated with the clinical prolongation of the QTc interval and, 3) a prolonged QTc interval does not always linearly correlate to the occurrence of TdP. The generally accepted explanation for these discrepancies is that although the inhibition of I_Kr_ plays a critical role in delaying repolarization, activation of inward late sodium (late I_Na_) and/or calcium currents (I_Ca_) have been shown to be needed to generate a proarrhythmic response (Nemec et al., 2016). As such, drugs that inhibit both inward (late I_Na_ and I_Ca_) and outward (I_K_) cardiac currents (*e.g.* ranolazine, verapamil) tend to prolong the QTc interval, but are not associated with the generation of TdP due to the blockade of inward currents (Fermini and Fossa, 2003; Fermini et al., 1995).

As a result, evaluation of cardiac proarrhythmic risk during drug development is evolving. The new emphasis is placed on assessing comprehensive drug-induced proarrhythmic risk rather than the traditional assessment of preclinical hERG blockade-related QTc interval prolongation. To that extent, “the comprehensive in vitro proarrhythmia assay” (CiPA) was established to develop a new paradigm for assessing proarrhythmic risk. One of the pillars of this initiative includes *in silico* integration of cellular electrophysiologic effects based on ionic current effects. *In silico* assays have a great potential to provide an integrative assessment of mixed cardiac ion channel inhibition as well as a high-throughput (HTS) assessment of proarrhythmic risk in early drug development. *In silico* modeling and simulation is beginning to be utilized more frequently to predict clinical proarrhythmic risk and also the outcome of preclinical *in vivo* or *ex vivo* models that generally are conducted later in drug development (Passini et al., 2017). Previous studies have evaluated the ability of *in silico* assays to predict the outcome of more resource intensive *in vivo* models such as drug effects on APD in isolated canine cardiomyocytes and in the rabbit ventricular wedge, respectively (Beattie et al., 2013; Davies et al., 2012).

Our current approach is analogous as it evaluates *in silico* simulations of action potential duration and electromechanical window (EMw) results (Morissette et al., 2016; Passini et al., 2019) to predict the QTc interval results in the cardiovascular anesthetized guinea pig assay (Morissette et al., 2013). Furthermore, this study also evaluates the usefulness of combining a mathematical translational pharmacokinetic/pharmacodynamic (tPKPD) model with the *in silico* qualitative endpoints to better predict the CVGP results. This tPKPD relationship could enable the prediction of unbound plasma concentration at which a new chemical entity (NCE) presents a QTc interval prolongation risk in the anesthetized CVGP assay. In turn, simulations using combined *in silico* and tPKPD models could help identify potential pro-arrrhythmic agents and determine the relevant concentration at which they may have a meaningful effect in animals or humans.

## 2 Methods

### 2.1 Statement on use and care of animals

All aspects of the animal use were in accordance with the Guide for the Care and Use of Laboratory Animals (Institute of Laboratory Animal Resources, Commission on Life Sciences, National Research Council, 2011) and approved by the Institutional Animal Care and Use Committee (IACUC) of Merck & Co., Inc., Kenilworth, NJ, USA.

### 2.2 PatchXpress® (PX) electrophysiology

#### 2.2.1 Measurement of I_Kr_ and I_Ks_ ion channel current activities: PatchXpress® automated patch clamp

Experimental procedures for measurement of I_Kr_ and I_Ks_ with PatchXpress® (PX) were previously described in detail (Trepakova et al., 2007, Zeng et al., 2008). In summary, whole-cell hERG or I_Ks_ currents were measured from heterologous human embryonic cells stably expressing hERG or I_Ks_ (KCNQ1/ KCNE1) channels using the automated PX system, PatchXpress® 7000A (Molecular Devices), at ambient temperature. Resistance of the planar patch plate chambers (SealChip_16_™ [AVIVA]) were between 1 and 3 MΩ. For measurement of hERG and I_Ks_, currents were elicited with their respective voltage-step protocol at 20-s inter-pulse intervals during control conditions and after addition of test compounds. The hERG current was elicited with the following voltage-step protocol: from a holding potential (Vh) of −80 mV, a brief 20-ms depolarizing prepulse to −50 mV was applied to obtain a baseline current followed by a return to Vh for 80 ms, an activating 4-s depolarizing step to a test potential (Vt) of +20 mV and a 4-s repolarizing step (repolarizing potential for deactivating tail current [Vtail]) to −50 mV.

The KvLQT1/KCNE1 current (I_Ks_) was elicited from a V_h_ of −50 mV with 3-s depolarizing steps to a V_t_ of +50 mV then followed by a 3-s repolarizing step (V_tail_) to −50 mV. Both hERG and I_Ks_ currents were quantified as peak deactivating tail current amplitude during V_tail_. Currents were monitored for stability for 5-min period before addition of the vehicle alone (DMSO control) or test agents diluted from stocks in 10 mM DMSO. Test agents were applied (60 μl) at sequentially increasing concentrations at a rate of 25 μl/s. For each condition or drug concentration, duplicate or triplicate 60 μl additions were made to each test well at 11-s intervals in order to achieve equilibrium and current was monitored for 5-min period at each condition or test agent concentration.

#### 2.2.2 I_Na_ channel activity measurements: PatchXpress automated patch clamp

A human cell line stably expressing the hNa_V_1.5, the electrophysiological recording solutions and the methods for the determination of activity on the cardiac Na^+^ channel were described previously (Penniman et al., 2010).

Briefly, whole cell hNa_v_1.5 currents (I_Na_) were recorded from isolated HEK-293 cells using 16-chamber planar glass electrodes (Sealchip™) and the whole-cell variant of the PX technique with the PatchXpress® 7000A automated PX (Molecular Devices, Sunnyvale, CA). SealChip ‘hole’ resistances were between 1 MΩ and 3 MΩ in the presence of the indicated recording solutions. Whole-cell hNav1.5 currents were low-pass filtered at a cut-off frequency of 3 kHz and digitally sampled at 15 kHz. hNav1.5 current was elicited using 30-ms pulses to −20 mV from a V_h_ of −100 mV and quantified using the amplitude of the negative (inward) peak current. Following vehicle or test article addition, voltage pulses were applied at a rate of 0.2 Hz for at least 6 min to allow equilibration of the current to a new steady state. Subsequently, a train of 60 pulses was applied at a rate of 3 Hz to determine rate-dependent effects of the test article. Typically, three ascending test article concentrations were tested sequentially in half-log increments on each cell. For each condition, *i.e.*, a given pulsing rate and test concentration, peak inward Na^+^ current amplitudes were quantified as the average of final three pulses in a train.

### 2.3 Measurement of MK-499 binding, I_CaL_ and I_Na_ channel activity by high throughput screening (HTS) methods

#### 2.3.1 MK-499 filter binding assay

This method is an *in vitro* test for the inhibition of ^35^S-MK-499 binding to the hERG channel expressed in HEK293 cells, known as hERG-1B/HEK293 cell line.

Cell membranes were prepared from HEK-293 cells constitutively expressing hERG. Cells were harvested and homogenized in Tris-EDTA buffer containing 50 mM Tris and 1 mM EDTA, pH 7.4. Homogenates were centrifuged at 45,000*g* for 50 min at 4 °C. The pellet was washed once in Tris-EDTA, centrifuged, resuspended at a concentration of 5 mg/ml in binding buffer containing (in mM) 70 NaCl, 60 KCl, 1 CaCl_2_, 2 MgCl_2_, and 10 HEPES, pH 7.4, and stored at −70 °C.

On the day of the assay, hERG membranes were thawed and diluted to a concentration of 180 μg/ml in binding buffer and 25 uL of membrane were added to each well of a 384 W deep well block (Axygen, P-384-120SQ-C). For competition binding studies, 1 ul of test compounds in DMSO at varying concentrations or DMSO alone (control) were added to the wells containing membrane. The binding reaction was initiated by adding 25 ul of ^35^S-labeled MK-499 (750–850 Ci/mmol, custom synthesis, Perkin Elmer) was added (achieving a final concentration of 50 pM). After incubation for 90 min at room temperature, the binding was stopped by filtration of membranes through a 384-well Multiscreen-HTS FC filter plate (Millipore, MZFCNOW50) that was prewet before filtration with 20 uL of prewet buffer (0.01% Poly(ethyleneimine) (Sigma, P3143), 0.01% Triton X-100 (SigmaUltra, T9284)). The filters were washed using a Biotek Elx405 plate washer that has been primed with cold 1× wash buffer (20× Wash Buffer: 1 M HEPES/NaOH, pH 7.4, 200 ml; 5 M NaCl, 520 mL; 1 M MgCl2, 40 mL; 1 M CaCl_2_, 20 mL; DH20, 220 mL). The assay mixture is transferred to the filter plate and then each assay plate well is washed with 30 uL/well of room temperature 1× wash buffer to ensure the assay mixture is completely removed. The filter plate was washed with 1× cold wash buffer with 100uL/well, twice. Washed filter plates were dried for at least 75 min in a 55 °C oven, and radioactivity associated with each filter was measured by the addition of 10 ul Microscint-0 (Perkin Elmer 6,013,611) scintillation liquid and counting in a 384-well scintillation counter (Topcount or MicroBeta, Perkin Elmer).

#### 2.3.2 Ca^2 +^ influx fluorescence (FLIPR) assay

Human L-type calcium channels (hCav1.2) composed of 3 calcium channel subunits (α_1C_, α_2_δ, ß_2a_) (Balasubramanian et al., 2009) were stably expressed in HEK-293 cells, along with an inwardly-rectifying potassium channel, hKir_2.3_, to set a more negative resting membrane potential (− 65 mV at an external K^+^ concentration,[K^+^]_e_, of 5.8 mM) and promote depolarization of the resting membrane potential by raising [K^+^]_e_ (Xia et al., 2004) and thereby increasing the K^+^ equilibrium potential. HEK-293 cells were grown in culture media containing DMEM (Gibco 11,960), without glutamine and sodium pyruvate, but with 4.5 g/L d-Glucose; supplemented with 10% fetal bovine serum, 100 U/ml penicillin, and 100 μg/ml streptomycin, as well as selection antibiotics Geneticin G418 (100–800 μg/ml) for α1C, Zeocin (40 μg/ml) for Kir2.3, and Hygromycin B (100–250 μg/ml) for β2a. HEK-Cav1.2 cells were incubated at 37 °C in filtered ambient air supplemented with 5% CO_2_. Cells were grown to approximately 70–80% confluency and passaged twice-a-week. All tissue culture reagents were obtained from ThermoFisher Scientific/LifeTechnologies.

Prior to conducting assays, cells were seeded in poly-d-lysine coated 384-well plates (BioCoat) at 60 K cells per well and incubated for ≤24 h at 37 °C. On the day of assay conduct, cells were rinsed with wash buffer (containing in mM: 5.8 KCl, 146.2 NaCl, 0.005 CaCl_2,_ 1.7 MgCl_2,_ and 10 HEPES) and then incubated in wash buffer supplemented with 4 μM FLUO-4 AM, 0.02% Pluronic acid (both from Molecular Probes), and 10 mM d-glucose for 30 min at room temperature in the dark. After this Ca^2 +^-sensitive dye loading step, cells were washed with and then kept in 0.05 ml incubation buffer (containing in mM: 25 KCl, 127 NaCl, 0.005 CaCl_2,_ 1.7 MgCl_2,_ and 10 HEPES) with test agent or positive or negative control drugs at various test concentrations for 30 min at room temperature in the dark. All buffers were adjusted to pH 7.5 with NaOH or HCL prior to use. The plate was subsequently placed in the reading chamber of a fluorimeter (FLIPR^TETRA^, Molecular Devices) and the emission of each well at a wavelength of 535 nM was measured in response to excitation at a wavelength 480 nM at sampling intervals of 1.6 s.

Following a baseline reading for 10–20 s, Ca^2 +^-trigger buffer (containing in mM: 119 NaCl; 25 KCl; 4 mM CaCl_2_; 1.7 MgCl_2_ and 10 HEPES) was added at a volume equal to that of the incubation buffer already present in the well, after which the emission intensity at 535 nM was measured for another 20–30 s. The Ca^2 +^ influx signal was quantified as the difference between the baseline and the peak of the emission signal in response to the addition of Ca^2 +^-trigger buffer, which was typically reached within 20 s thereafter. The Ca^2 +^ signal from each well containing test agent was normalized to a signal window defined as the difference between the positive (100% inhibition) and negative (0% inhibition) control signals.

#### 2.3.3 hNa_V_1.5 FLIPR assay

The HEK-hNa_V_1.5 cell line is a stably transfected HEK-293 cell line over expressing hNav1.5. Cells were grown in culture media containing MEM (Gibco 3230–026), supplemented with 10% fetal bovine serum (Hyclone SH30070.02), 1% penicillin/streptomycin/l-Glutamine (Gibco 10,378–016), 1% non-essential amino acids (Gibco 11140-035). HEK-hNa_V_1.5 cells were incubated at 37 °C with 5% CO_2_. Cells were grown to approximately 70–80% confluency and passaged twice a week.

Prior to conducting assays, cells were seeded in poly-d-lysine coated 384-well plates (BD Biosciences 356,697) at 15 K cells per well and incubated for 48 h at 37 °C.

On the day of assay, cells were washed with assay buffer (containing in mM: 4.5 KCl, 165 NaCl, 2 CaCl_2,_ 1 MgCl_2,_ 10 glucose, and 10 HEPES) and then incubated in 25 ul 1× blue dye solution (Molecular Devices BLUE R8042 diluted in assay buffer). Test agent or positive or negative control drugs at various test concentrations were added and incubated for a 30-min period at room temperature in the dark. The plate was subsequently placed in the reading chamber of a fluorimeter (FLIPR^TETRA^, Molecular Devices) and the emission of each well at a wavelength of 565 nM was measured in response to excitation at a wavelength 510 nM at sampling intervals of 3.5 s for 60 reads. Following a baseline read, 25 ul of 60uM veratridine (Sigma-Aldrich V5754–25 MG) prepared in assay buffer was added, after which the emission intensity at 565 nM was measured for another 60 reads. The signal was quantified as the difference between the baseline and the peak of the emission signal in response to the addition of Veratridine, which was typically reached within 20 s thereafter. The Ca^2 +^ signal from each well containing test agent was normalized to a signal window defined as the difference between the positive (100% inhibition) and negative (0% inhibition) control signals.

### 2.4 Anesthetized Guinea pig assay

Details of the anesthetized CVGP assay have been described previously in Morissette et al. (2016). Briefly, male Hartley guinea pigs (BW: 350**–**500 g) were anesthetized with a bolus mixture of ketamine/xylazine (85/5 mg kg^− 1^, IM) then an intravenous (iv) infusion of ketamine/xylazine (40/0.5 mg kg^− 1^ h^− 1^, to effect). The jugular vein and left carotid arteries were cannulated for test article administration and arterial blood pressure (BP) monitoring and blood collection, respectively. The body surface electrocardiogram (ECG) was measured *via* subcutaneous needle electrodes to record modified lead-II ECG. After surgery, anesthetized GPs were stabilized for a minimum of 10 min before beginning baseline data collection. Solutions of 3 varying test-article concentrations suitable for administering selected doses were prepared in appropriate vehicles. Vehicle alone or test-article solutions were administered i.v. with each dose level infused over a 20-min period. Each test article and vehicle were evaluated in ≥ *n* = 3 male anesthetized GPs. Blood sampling for pharmacokinetic analysis was taken at 10 and 19 min of each dose level.

### 2.5 *In Silico* simulation

A control population of 107 human ventricular action potential (AP) models was constructed using the O'Hara-Rudy dynamic (ORd) model (O'Hara et al., 2011) and the experimentally-calibrated population of models methodology (Britton et al., 2013; Passini et al., 2017). A description of the design of this control population of models is described in Passini et al., 2019 (Passini et al., 2019).

All simulations in this study were conducted using the Virtual Assay (v.1.3.6402014 Oxford University Innovation Ltd. Oxford, UK), a C^++^ based software package for *in silico* drug assays (Passini et al., 2017).

Drug effects were simulated using a simple pore-block model (Brennan et al., 2009), with IC_50’s_ acquired internally using whole-cell automated PX (PatchXpress ®) and/or HTS methods for 4 ion channels: fast Na^+^ current (I_Na_) and rapid/slow delayed rectified K^+^ current (I_Kr_/I_Ks_), and L-type Ca^2+^ current (I_CaL_). *In Silico* data was generated by either using the combination of IC_50’s_ from PX (I_Na_ and I_Kr_/I_Ks_) and the Ca^2 +^ influx fluorescence assay (I_CaL_) or the combination of the HTS IC_50’s_ generated using MK-0499 Filter Binding Assay (I_Kr),_ the Ca^2 +^ FLIPR (I_CaL_) and the hNa_V_1.5 FLIPR Assay.

**Fig. 1:**
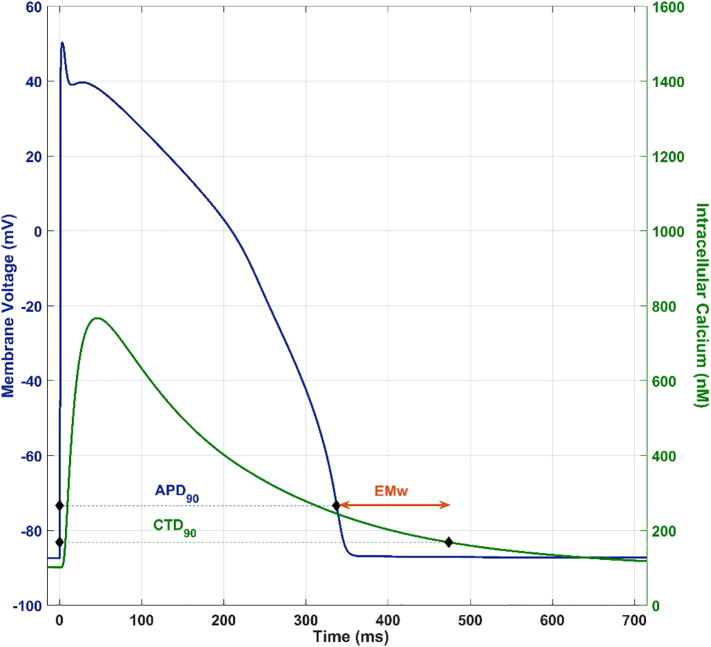
*In silico* APD_90_ and EMw. The EMw is defined here as the difference between the Ca^2+^ Transient Duration at 90% repolarization (CTD_90_) and the action potential duration at 90% repolarization (APD_90_).

Multiple concentrations of each compound were investigated (0, 0.03, 0.1, 0.3, 1, 3 and 10 μM) in the 107-simulated cell model. These concentrations typically cover a wide enough range that is relevant and sufficiently above (>30-fold) the projected free clinical concentrations of the selected NCEs. Virtual cells were paced at 1 Hz for 500 beats and the last AP and Ca^2+^ transient traces of each simulation were compared with the corresponding control (500 beats at 1 Hz in the absence of drug). All AP traces were checked for repolarization and depolarization abnormalities. For cell models not displaying abnormalities, the average action potential duration at 90% repolarization (APD_90_) and the Ca^2+^ transient duration at 90% of repolarization (CTD_90_) were computed, along with a single cell approximation of the *in vivo* EMw, defined as the difference between Ca^2+^ transient duration at 90% (CTD_90_) and APD_90_, as shown in Fig. 1 (Passini et al., 2019).

### 2.6 Translational pharmacokinetic and pharmacodynamic (tPKPD) M5 pruned model

M5 tree is a decision tree model that allows to develop classification systems that predict or classify future observations based on a set of decision rules. It has been developed for dealing with regression task and operates on continuous numerical dependent variable (Quinlan, 1992). In general, they follow the classification and regression tree (CART) approach, but the leaf node constant value has been replaced by fitted multivariate linear regression model. One of the features of the M5 trees is automatic pruning (M5P) that allows for effective tree size reduction. For each internal node, the algorithm compares the estimated error of that node and estimated error of a subtree below the node. In the situation when the subtree does not provide added performance value of the whole tree, it is cut off. M5P trees have been widely used in multiple areas and various problems (Freitas et al., 2015). The M5P building algorithm implemented in the Waikato environment for knowledge analysis (WEKA) was used during the study. A similar approach was previously used to analyze cardiac safety of antituberculotic drugs (Polak et al., 2018).

The model development was preceded by the data pre-processing. The above described *in silico* generated data that were used as they stand, however the *in vitro* measured IC_50_ values were re-calculated from nanomolar (nM) to micromolar (μM) and negative logarithm (pIC_50_) was calculated. The output declared as tPKPD QTc EC_5_ was recalculated to the logarithmic value (see Table 5).

The model development was based on the leave-one-out (LOO) methodology and automatic pruning. The former means that the algorithm of automatic tree building was restarted n times (where “n” – number of compounds in original learning dataset) and every time 1 compound was excluded to serve as internal validation. An identical methodology was applied to develop multiple linear regression-based models, which were further used as the comparators and point of reference for the MP5 based models. In each case linear regression-based models' performance was lower as compared against the model of choice – M5P trees.

The original input dataset consisted of 12 *in silico* computed parameters and 7 (‘PX’ approach) or 3 (‘HT’ approach) *in vitro* measured data (Table 1). The automated pruning algorithm reduced the input vectors as presented in Table 2.

**Table 1:** *In vitro* data used as the input information for the M5P-based model in two tested approaches PX and HTS, respectively.

Approach parameter (pIC50)	PX	HTS
High Throughput MK-499 Binding		×
High Throughput hNav1.5		×
High Throughput ICa	×	×
PatchXpress INa (hNav1.5)	×	
PatchXpress INa (hNav1.5) Hill Coefficient	×	
PatchXpress I_Kr_ (hERG)	×	
PatchXpress I_Kr_ (hERG) Hill Coefficient	×	
PatchXpress I_Ks_ (KCNQ1/ KCNE1)	×	
PatchXpress I_Ks_ (KCNQ1/ KCNE1) Hill Coefficient	×

**Table 2:** Final input parameters specific for PX and HTS.

QTc	
‘PX’	‘HTS’
PatchXpress I_Kr_ (hERG) IC_50_	High Throughput MK-499 Binding IC_50_
PatchXpress I_Kr_ (hERG) Hill Coeff.	High Throughput hNav1.5 IC_50_
High Throughput I_Ca_ IC_50_	High Throughput I_Ca_ IC_50_
PatchXpress hNav1.5 IC_50_	Average maximum upstroke velocity (dV/dt_MAX) for all simulated cells at the 3rd highest concentration tested
Average resting membrane potential (RMP) for all simulated cells at the 4th highest concentration tested	
Average resting membrane potential (RMP) for all simulated cells at the 5th highest concentration tested	
Average peak voltage (Vpeak) in simulated cells at 5th highest concentration tested	
Average Ca^2+^-transient duration at 50% (CTD_50_) of the initial base value for all simulated cells at the 2nd highest concentration tested	
Average action potential duration at 50% (APD_50_) repolarization for all simulated cells at the third highest concentration tested	

## 3 Results

### 3.1 Ion Channel, *In Silico* and anesthetized CVGP assay data

A general workflow to determine the predictive capacity of the *in silico* model alone or the *in silico* model in combination with a tPKPD model to predict the CVGP QTc interval prolongation results is presented in the supplementary section. In summary, the ion channel inhibition data was generated for *n* = 73 NCEs using PX and *n* = 51 of the same set of NCEs using HTS methods (Table 3). The NCEs (all small molecules) were from various chemical classes which cover 5 therapeutic areas and over 41 different mechanisms of action. The ion channel IC_50s_ are used as inputs for the *in silico* model which simulates the effects at varying NCE concentrations on the virtual ventricular action potentials and electromechanical window. The qualitative directional effect on the simulated APD_90_ and EMw are reported in Table 5 along with the tPKPD predicted and the actual CVGP unbound concentrations at which we observed a 5% increase in QTc interval in the CVGP (QTc EC_5_). Using the qualitative directional effects on the simulated APD_90_ and EMw enables the determination of the predictive capacity of the *in silico* model alone to prolong the QTc interval in the CVGP (described in section 3.3). In general, compounds that prolong the QTc interval and the action potential, respectively in the CVGP and *in silico* assays, will also shorten the EMw *in silico*. NCEs that are mixed ion channel inhibitors typically do not prolong the QTc interval in the CVGP assay and do not shorten the simulated EMw, *in silico*. Also, NCEs that have hCaV1.2 IC_50s_ which are within 2-fold of the hERG or MK-499 IC_50_ don't typically prolong the CVGP QTc interval or simulated action potential and tend to prolong the simulated EMw.

**Table 3:** Ion channel inhibition data from the PX and HTS platforms.

NCE	PX hERG IC_50_ (μM)	PX hNav1.5 IC_50_ (μM)	PX I_Ks_ IC_50_ (μM)	HTS hCav1.2 IC_50_ (μM)	HTS MK-499 IC_50_ (μM)	HTS hNav1.5 IC_50_ (μM)
1	0.01	8.3	ND	3.25	0.01	43
2	0.95	9.4	270	ND	NA	NA
3	26.5	ND	ND	ND	26	NA
4	1	0.6	100	ND	NA	NA
5	0.007	6.7	ND	3	1.1	5.3
6	2.6	11	5.9	0.1	NA	NA
7	42	ND	ND	ND	NA	NA
8	12	68	5.7	7.4	3.1	12.4
9	36	15	79	16.2	9.9	0.5
10	18	14	ND	ND	NA	NA
11	8.2	48	100	12.6	24.7	ND
12	27	68	150	ND	NA	NA
13	0.86	100	ND	5.1	0.3	3.5
14	27	75	ND	13.3	10.8	4.7
15	55	ND	ND	ND	53	ND
16	25	ND	ND	ND	NA	NA
17	15	43	21	16.9	32.6	39
18	21	94	ND	13.4	34.3	12.9
19	40	45	6	27	2.2	ND
20	27	ND	ND	79	49	ND
21	11	19	13	ND	NA	NA
22	26	43	150	35	41.6	15.2
23	31	45	ND	ND	NA	NA
24	6.4	100	ND	11	47	14
25	8.1	36	ND	ND	NA	NA
26	0.58	11	44	ND	NA	NA
27	50	ND	ND	ND	38	ND
28	14	ND	ND	21.4	34.9	ND
29	40	50	ND	2.8	46	136
30	11	ND	ND	ND	32.3	ND
31	22	ND	ND	ND	33	ND
32	23	36	ND	ND	NA	NA
33	48	4.1	38	19	5.7	29.4
34	22	55	ND	7	41.9	23.4
35	26	ND	ND	21.5	31.2	ND
36	4.5	16	63	ND	NA	NA
37	29	ND	ND	115	39	29
38	12	42	ND	6.9	6.4	10.9
39	10	94	ND	27.9	10.4	19.2
40	21	19	ND	43	56.5	ND
41	8.5	38	ND	ND	NA	NA
42	26	4	15	16.4	11.1	12.7
43	35	ND	83	18.6	50	ND
44	47	21	29	13.8	18.4	28.8
45	58	ND	ND	ND	28	ND
46	5.3	ND	ND	107	10.9	94
47	12	36	115	18.4	19.8	15.1
48	56	68	48	28.7	47.2	88
49	44	60	ND	9.1	ND	83
50	82	53	ND	ND	NA	NA
51	0.95	45	ND	17.5	3.6	ND
52	19	150	83	ND	6.3	31.8
53	5.4	ND	ND	300	NA	NA
54	2.5	60.9	15	3.12	NA	NA
55	2.1	23	300	0.785	1.7	5.6
56	1	70	106	3	16.1	ND
57	4.8	0.94	300	ND	26.7	ND
58	0.14	638	31	2.3	0.42	2.8
59	0.33	7.5	95	10	NA	NA
60	25	ND	ND	26	NA	NA
61	1.8	17	14	4.9	3	ND
62	4.3	15	3.7	0.47	3.8	31
63	9.1	90	300	0.195	3.8	7.5
64	1.5	300	300	4.1	ND	ND
65	1.1	12	60	1.3	3.6	ND
66	2.2	300	300	1.75	3.3	20.3
67	0.14	ND	ND	ND	NA	NA
68	4	ND	ND	19.04	22.9	19.5
69	2	ND	ND	20.4	ND	ND
70	5	53.3	300	ND	19.5	ND
71	11	13.5	ND	3.087	NA	NA
72	83	35	ND	ND	NA	NA
73	3.3	6.9	13.6	1.251	NA	NA

ND: An IC_50_ was not determined, no meaningful inhibition (<10% inhibition) was observed at the highest tested concentration (30 μM), NA: No data is available: the NCE was not tested.

Combining the *in silico* model results with the predicted QTc EC_5_ (Table 5) from the tPKPD model enables the re-evaluation of the predictive capacity (Fig. 3C and D) of the *in silico* model results in combination with the tPKPD model to prolong the QTc interval in the anesthetized CVGP (described in section 3.4). In general, this will reclassify several compounds that were identified as false positives given it is now possible to relate the predicted QTc EC_5_ to the actual concentration achieved in the anesthetized CVGP.

### 3.2 Probabilistic assessment of QTc prolongation in the anesthetized CVGP assay using hERG or MK-499 inhibition data alone

**Fig. 2:**
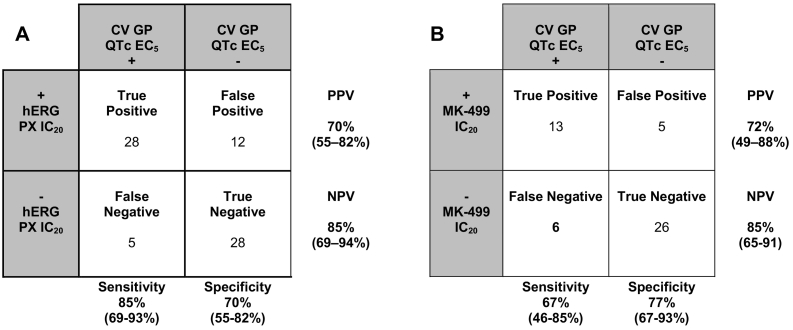
Matrix results (with 95% confidence intervals indicated) of hERG and MK-499 IC_20_ values relative to the anesthetized CVGP QTc EC_5_ using ion channel data generated using A) the PX platform (*n* = 73), and B) the HTS platform (*n* = 51). PPV: Positive predictive value, NPV: Negative predictive value.

Confusion matrix analysis (Fig. 2) was conducted using either the PX hERG or MK-499 IC_20_'s as compared to the CVGP QTc EC_5_ concentrations for each NCE (Table 2). NCE's were qualified as “true positive” (TP) to prolong the QTc interval in the CVGP if the hERG or MK-499 IC_20_'s was lower or within 5-fold as compared to the CVGP QTc EC_5_. If the PX hERG or MK-499 IC_20_'s was >5-fold than the CVGP QTc EC_5,_ they were qualified as “false negative” (FN). NCE's were qualified as “true negative” (TN) if their hERG or MK-499 IC_20_ was larger than the highest free concentration achieved in the CVGP for which QTc interval prolongation was not observed. In these instances, the CVGP QTc EC_5_ was not defined and was listed as being greater than the highest free plasma concentration tested in Table 4. NCE's were qualified as “false positive” (FP) if the PX hERG or MK-499 IC_20_'s was lower than the highest concentration achieved in the CVGP without QTc interval prolongation.

**Table 4:** Confusion matrix results comparing the PX hERG IC_20_ or HTS MK-499 binding IC_20_ to the CVGP QTc EC_5_.

Compound	Guinea pig QTc EC_5_ (μM)^a^	PX hERG IC_20_ (μM)	PX hERG IC_20_ Confusion matrix result PX	HTS MK-499 IC_20_ (μM)	HTS MK-499 IC_20_ (μM) Confusion matrix result HTS
1	0.07	0.004	TP	0.003	TP
2	1.2	0.2	TP	NA	NA
3	1.8	12	FN	9.2	FN
4	1.6	0.4	TP	NA	NA
5	0.02	0.0028	TP	0.3	FN
6	>3	1.04	FP	NA	NA
7	15	15.2	TP	NA	NA
8	>0.7	4.8	TN	0.8	TN
9	>2	12	TN	1.9	FP
10	1.6	8.1	FN	NA	NA
11	>5	2.6	FP	8.2	TN
12	>8.7	7.5	FP	>30	TN
13	1	0.2	TP	0.1	TP
14	>5.4	12	TN	3.5	FP
15	>20	21	TN	19.2	FP
16	10	8.7	TP	NA	NA
17	>5	5.5	FP	12.4	TN
18	>0.385	8.4	TN	12.6	TN
19	>0.05	16	TN	0.82	FP
20	>7.7	9.2	TN	18.5	TN
21	>0.1	4.3	TN	NA	NA
22	>0.89	10.1	TN	15.4	TN
23	5	17	FN	ND	NA
24	7.2	1.5	TP	15.2	TP
25	1.2	6.5	FN	ND	NA
26	1	0.2	TP	NA	NA
27	>4.5	21	TN	14.2	TN
28	>4.6	4.2	FP	11.1	TN
29	>0.14	12	TN	16.2	TN
30	4	3.5	TP	8.2	TP
31	>2	8	TN	12.5	TN
32	6	7.5	TP	NA	NA
33	>0.1	16	TN	1.6	TN
34	>16	7.2	FP	14.8	FP
35	> 45.8	8.4	FP	12.48	FP
36	3.5	1.5	TP	NA	NA
37	>2.5	10.8	TN	14.2	TN
38	>60	3.5	FP	2.4	FP
39	0.9	3.5	TP	4.1	TP
40	1.4	7.6	FN	18.5	FN
41	>4.4	2.5	FP	NA	NA
42	>0.27	8.3	TN	3.5	TN
43	>5.5	12	TN	18.2	TN
44	>1.98	16	TN	6.5	TN
45	44	19.4	TP	10.5	TP
46	0.988	2.5	TP	3.8	TP
47	>0.2	3.6	TN	6.4	TN
48	>4.7	20.6	TN	16.2	TN
49	>0.3	15.4	TN	6.8	TN
50	>31	33.3	TN	NA	NA
51	0.3	0.2	TP	1.1	TP
52	2.8	5.4	TP	2.1	TP
53	0.4	2.2	TP	NA	NA
54	>0.4	0.8	TN	NA	NA
55	>1	0.56	FP	0.68	FP
56	1.8	0.2	TP	6.44	TP
57	2	1.4	TP	10.1	FN
58	0.23	0.056	TP	0.168	TP
59	>3.3	0.132	FP	NA	NA
60	19.9	8.2	TP	NA	NA
61	0.1	0.34	TP	0.8	FN
62	>0.2	3.4	TN	1.1	TN
63	>0.4	5.2	TN	1.3	TN
64	> 0.07	0.8	TN	ND	TN
65	1	0.33	TP	1.1	TP
66	>3.4	0.56	FP	0.9	FP
67	0.3	0.05	TP	NA	NA
68	3	1.1	TP	8.2	TP
69	0.5	0.6	TP	NA	NA
70	1.2	1.7	TP	6.5	FN
71	>2.3	6.2	TN	NA	NA
72	>5.4	28.4	TN	NA	NA
73	>0.8	1.4	TN	NA	NA

TP: True Positive, TN: True Negative, FP: False Positive, FN: False Negative, ND: An IC_50_ was not determined, no meaningful inhibition was observed at the highest tested concentration (30 μM), NA: No data is available: the NCE was not tested.

a The listed concentrations are free plasma concentrations.

Using this set of rules, it was determined that specificity is similar using either PX or MK-499 binding data to predict QTc interval prolongation in the CVGP. However, the rate of false negatives using PX hERG inhibition data was lower as compared to when MK-499 binding data was used which resulted in higher sensitivity with the PX data.

### 3.3 Probabilistic assessment of QTc interval prolongation in the anesthetized CVGP assay using the qualitative outcome of *in silico* simulated APD prolongation and EMw shortening

**Fig. 3:**
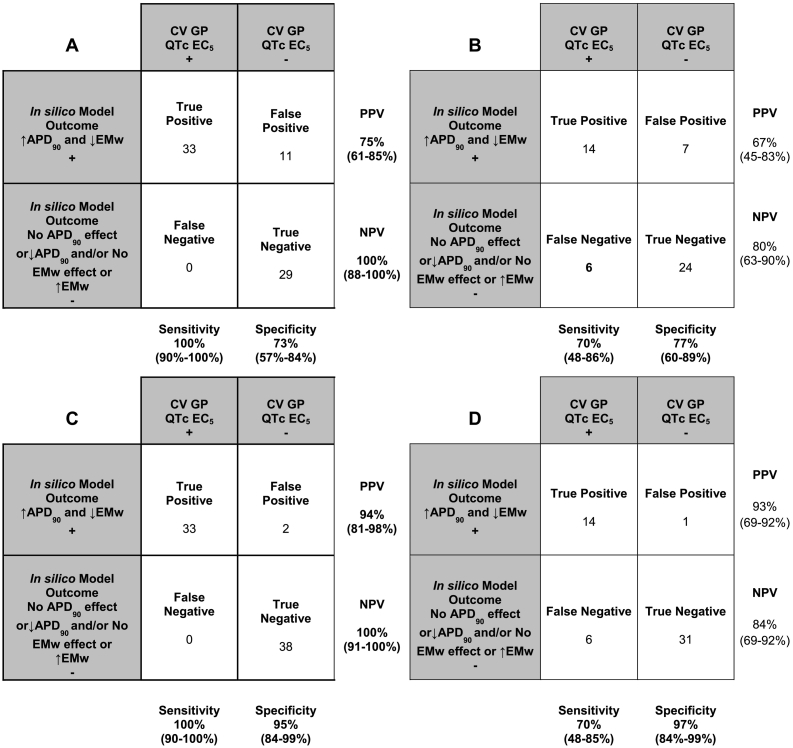
Matrix results (with 95% confidence intervals indicated) using the *in silico* model endpoints (APD_90_ and EMw) relative to the CVGP QTc EC_5_ using *in vitro* ion channel data obtained from PX (A) or HTS (B) and matrix results when adjusted with tPKPD model projected QTc EC_5_ using PX (C) or HTS (D) ion channel data. PPV: Positive predictive value, NPV: Negative predictive value.

Effects on simulated APD_90_ and EMw using either HTS (*n* = 51 NCEs) or PX ion channel data (*n* = 73 NCEs) was computed at various drug concentrations (6 concentrations per NCE) and their qualitative results were compared to the CVGP QTc EC_5_ interval effect. Confusion matrix analysis was conducted to assess the concordance of the *in silico* results using PX (Fig. 3A) or HTS data (Fig. 3B) to predict QTc interval prolongation in the CVGP. NCEs were qualified as “positive” *in silico* to prolong the QTc interval in the CVGP when both the APD_90_ prolonged and the EMw shortened from baseline over the range of tested concentrations. However, if the simulated APD_90_ did not prolong and/or the EMw did not shorten (<5% change over the range of concentrations tested), the NCE was considered “negative” to prolong the QTc interval in the CVGP assay. In the CVGP, a compound was considered “positive” if it prolonged the QTc interval over the range of tested concentrations and a CVGP QTc EC_5_ was defined. If there was no QTc prolongation in the CVGP the NCE was defined as “negative” *in vivo* and the CVGP QTc EC_5_ was listed in Table 5 to be greater than the highest free plasma concentration tested in the CVGP. As such, a TP NCE was identified if both the simulated APD_90_ prolonged and the simulated EMw shortened and a CVGP QTc EC_5_ was defined. A TN NCE was defined if no CVGP QTc EC_5_ was identified and the APD_90_ shortened or the EMw prolonged. The confusion matrix results for each individual NCE are presented in Table 5 and applied in Fig. 3. Higher sensitivity to predict QTc interval prolongation in the CVGP is achieved when using the *in silico* model outcomes generated with PX ion channel data as compared to when using HTS ion channel data. Specificity is similar when using PX or MK-499 data as inputs for the *in silico* model.

**Table 5:** Confusion matrix results using qualitative outcomes from *in silico* simulated endpoints or when adjusted with tPKPD QTc EC_5_ projected concentrations.^a^

Compound	CVGP QTc EC_5_ (μM)^a^	Data generated using PX ion channel data					Data generated using HTS ion channel data				
		*In Silico* APD_90_ directional effect	*In Silico* EMw directional effect	Confusion matrix result using only *in silico* results	tPKPD QTc EC_5_ (μM)^a^	Confusion matrix result adjusted with tPKPD results	*In Silico* APD_90_ directional effect	*In Silico* EMw directional effect	Confusion matrix result	tPKPD QTc EC_5_ (μM)^a^	Confusion matrix result adjusted for tPKPD results
1	0.07	↑	↓	TP	0.03	TP	↑	↓	TP	0.08	TP
2	1.2	↑	↓	TP	2.4	TP	NA	NA	NA	NA	NA
3	1.8	↑	↓	TP	1.9	TP	↑	↓	TP	4.8	TP
4	1.6	↑	↓	TP	1.5	TP	NA	NA	NA	NA	NA
5	0.02	↑	↓↑	TP	0.02	TP	↑	↓	TP	0.21	FN
6	>3	↑	↑	TN	2.9	TN	NA	NA	NA	NA	NA
7	12	↑	↓	TP	15	TP	NA	NA	NA	NA	NA
8	>0.7	↑	↑	TN	1.3	TN	↑	↑	TN	0.5	TN
9	>2	↑	↑	TN	1.9	TN	↑	↑	TN	5.7	TN
10	1.6	↑	↓	TP	6.01	TP	NA	NA	NA	NA	NA
11	>5	↑	↑	TN	3.1	TN	↑	↑	TN	0.3	TN
12	>8.7	↑	↓	FP	9.2	TN	↑	↑	TN	468	TN
13	1	↑	↓	TP	0.4	TP	0.03	0.03	TP	0.1	TP
14	>5.4	↑	↑	TN	10.9	TN	↑	↑	TN	2.5	TN
15	>20	↑	↓	FP	28	TN	↑	↓	FP	32	TN
16	10	↑	↓	TP	19.9	TP	NA	NA	NA	NA	NA
17	>5	↑	↑	TN	3.4	TN	↑	↑	TN	3	TN
18	>0.385	↑	↑	TN	17	TN	↑	↑	TN	4.3	TN
19	>0.05	↑	↑	TN	2	TN	↑	↓	FP	0.08	TN
20	>7.7	↑	↓	FP	6.1	FP	↑	↓	FP	1.1	FP
21	>0.1	↑	↓	FP	5.2	TN	NA	NA	NA	NA	NA
22	>0.89	↑	↑	TN	2.2	TN	↑	↑	TN	6.9	TN
23	5	↑	↓	TP	9.1	TP	NA	NA	NA	NA	NA
24	7.2	↑	↓↑	TP	1	TP	↑	↑	FN	4.9	FN
25	1.2	↑	↓	TP	5.1	TP	NA	NA	NA	NA	NA
26	1	↑	↓	TP	1.6	TP	NA	NA	NA	NA	NA
27	>4.5	↑	↓	FP	4.7	TN	↑	↓	FP	6.4	TN
28	>4.6	↑	↑	TN	1.4	TN	↑	↑	TN	0.5	TN
29	>0.14	↓	↑	TN	12	TN	↓	↓	TN	1.2	TN
30	4	↑	↓	TP	14.1	TP	↑	↓	TP	5.6	TP
31	>2	↑	↓	FP	3.6	TN	↑	↓	FP	5.7	TN
32	6	↑	↓	TP	9.6	TP	NA	NA	NA	NA	NA
33	>0.1	↑	↑	TN	4.2	TN	↑	↓	FP	0.9	TN
34	>16	↑	↑	TN	18	TN	↑	↑	TN	3.1	TN
35	> 45.8	↑	↑	TN	5.6	TN	↑	↑	TN	0.5	TN
36	3.5	↑	↓	TP	3.6	TP	NA	NA	NA	NA	NA
37	>2.5	↑	↓	FP	3	TN	↑	↓	FP	8.5	TN
38	>60	↑	↑	TN	1.2	TN	↑	↑	TN	0.9	TN
39	0.9	↑	↓	TP	1.2	TP	↑	↓	TP	2	TP
40	1.4	↑	↓	TP	2	TP	↑	↑	FN	0.9	FN
41	>4.4	↑	↓	FP	5.2	TN	NA	NA	NA	NA	NA
42	>0.27	↑	↑	TN	1.9	TN	↑	↑	TN	1.9	TN
43	>5.5	↑	↑	TN	2.1	TN	↑	↑	TN	0.6	TN
44	>1.98	↑	↑	TN	2.4	TN	↑	↑	TN	2	TN
45	44	↑	↓	TP	29	TP	↑	↓	TP	5	TP
46	0.988	↑	↓	TP	1	TP	↑	↓	TP	2	TP
47	>0.2	↑	↑	TN	1.3	TN	↑	↑	TN	3	TN
48	>4.7	↑	↑	TN	2.7	TN	↑	↑	TN	1	TN
49	>0.3	↑	↑	TN	4.2	TN	↓	↑	TN	147	TN
50	>31	↑	↑	TN	134	TN	NA	NA	NA	NA	NA
51	0.3	↑	↓	TP	0.5	TP	↑	↓	TP	0.07	TP
52	2.8	↑	↓	TP	5.2	TP	↑	↓	TP	12.5	TP
53	0.4	↑	↓	TP	1	TP	NA	NA	NA	NA	NA
54	>0.4	↑	↑	TN	0.6	TN	NA	NA	NA	NA	NA
55	>1	↑	↑	TN	0.4	TN	↑	↑	TN	0.16	TN
56	1.8	↑	↓	TP	0.5	TP	↑	↑	FN	0.12	FN
57	2	↑	↓	TP	1	TP	↑	↓	TP	4.1	TP
58	0.23	↑	↓	TP	0.2	TP	↑	↓	TP	0.12	TP
59	>3.3	↑	↓	FP	0.2	FP	NA	NA	NA	NA	NA
60	19.9	↑	↓	TP	10	TP	NA	NA	NA	NA	NA
61	0.1	↑	↓	TP	0.5	TP	↑	↑	FN	0.04	FN
62	>0.2	↑	↑	TN	0.4	TN	↑	↑	TN	0.12	TN
63	>0.4	↓↑	↑	TN	0.4	TN	↑	↑	TN	0.12	TN
64	> 0.07	↑	↓	FP	0.5	TN	↓	↑	TN	18.9	TN
65	1	↑	↓	TP	0.2	TP	↑	↓	TP	0.1	TP
66	>3.4	↑	↑	TN	0.5	TN	↑	↑	TN	0.3	TN
67	0.3	↑	↓	TP	0.4	TP	NA	NA	NA	NA	NA
68	3	↑	↓	TP	0.7	TP	↑	↑	FN	3.1	FN
69	0.5	↑	↓	TP	0.6	TP	↓	↑	FN	37	FN
70	1.2	↑	↓	TP	1	TP	↑	↓	TP	3.8	TP
71	>2.3	↑	↑	TN	4.2	TN	NA	NA	NA	NA	NA
72	>5.4	↑	↓	FP	34	TN	NA	NA	NA	NA	NA
73	>0.8	↑	↑	TN	1	TN	NA	NA	NA	NA	NA

↑: Increase at tested concentrations, ↓ decrease at tested concentrations, ↓↑ decrease followed by an increase at higher concentrations, TP: True Positive, TN: True Negative, FP: False Positive, FN: False Negative, ND: IC_50_ was not determined, no meaningful inhibition (<10% inhibition) was observed at the highest tested concentration (30 μM), NA: No data is available, the NCE was not tested.

a The listed concentrations are free plasma concentrations.

### 3.4 Probabilistic assessment of QTc interval prolongation in the anesthetized CVGP assay when adjusted with the tPKPD model results

**Fig. 4:**
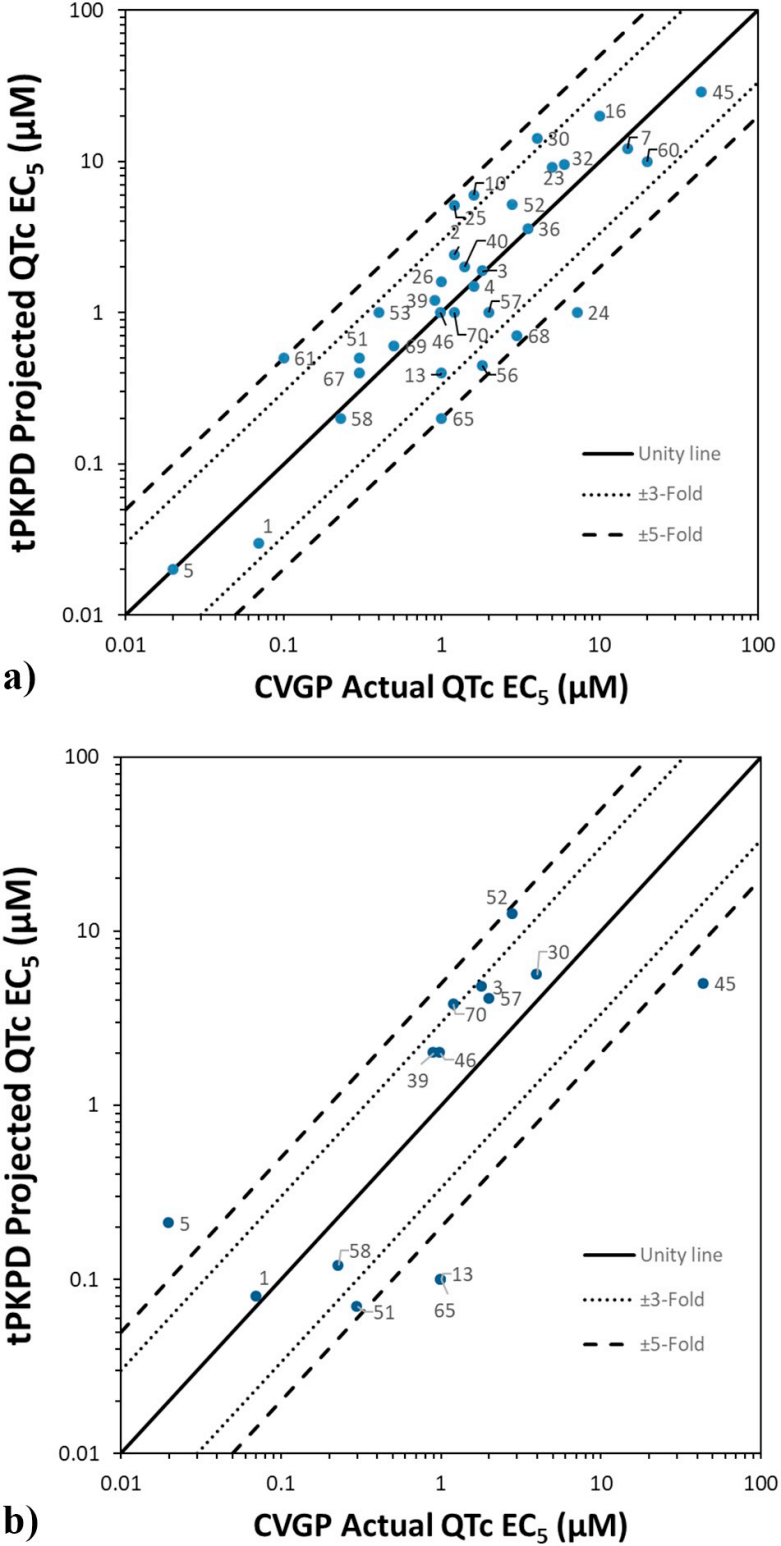
Translational Pharmacokinetic/Pharmacodynamic (tPKPD) results: A) tPKPD projected QTc EC_5_ as compared to actual CVGP QTc EC_5_ when using PX ion channel data. B) tPKPD projected QTc EC_5_ as compared to the actual CVGP QTc EC_5_ when using high throughput (HTS) ion channel data. Data points are labeled with their NCE number found in Table 3, Table 4, Table 5.

The concentration at which a CVGP QTc EC_5_ would occur was evaluated using either the HTS or PX ion channel data in a M5-pruned tPKPD model. The correlation between the projected free plasma concentration that caused a 5% prolongation in QTc interval with the HTS or PX tPKPD models and the actual plasma concentration that caused a 5% increase in QTc interval in the CVGP were plotted for all the NCEs that were qualified as positive in section 3.3 (Fig. 4A and B). In general results are within 5-fold of the unity line.

Positive NCEs (TP and FP) were further analyzed using the tPKPD results and confusion matrices are presented in Fig. 3B and D for HTS and PX data, respectively. However, all negative NCEs identified using the *in silico* model were considered negative and, as such, not further analyzed using the tPKPD model. All the TP compounds identified in section 3.3 stayed positive if the tPKPD model predicted QTc EC_5_ concentrations within 5-fold of the actual CVGP QTc EC_5._ In addition, if the tPKPD model predicted a QTc EC_5_ lower than the actual CVGP QTc EC_5_, the NCE was judged TP. In contrast, if a FP's predicted concentration to prolong the QTc interval is greater than the free concentration achieved in the CVGP where there is no QTc interval increase, the compound was re-classified TN. However, a FP stayed FP (compound 20 and 59 for PX and compound 20 for HTS) if the tPKPD QTc EC_5_ was lower than the highest CVGP tested free plasma concentration at which there was no QTc interval prolongation. The confusion matrix results adjusted with the tPKPD projections for each individual NCE are presented in Table 5. In summary, Fig. 3B and D show that when the tPKPD model is applied in combination with *in silico* modeling, a meaningful increase in specificity is observed for both PX (73 to 95%) or HTS (77 to 97%) ion channel inputs.

## 4 Discussion

Historically, assessment of potential cardiac conduction and repolarization liabilities for NCE in early drug discovery research relied heavily, and in some cases exclusively, on biochemical and/or functional *in vitro* ion channel studies. Often, the first *in vivo* data gathered for a NCE was in anesthetized non-rodent or conscious, telemetered non-rodent models. While telemetered, conscious, non-rodent models are considered the “gold standard” and a required step prior to first in human (FIH) studies, the cost, complexity and resources required have often pushed these evaluations later in the drug development process; creating a gap of knowledge on these important ECG risk liabilities in the lead optimization process. The utilization of *ex vivo* models (Di Diego et al., 2013; Picard et al., 2006; Valentin et al., 2004) and, perhaps more so, small animal *in vivo* models (Morissette et al., 2016; Schmitz et al., 2016) has enabled resource sparing, translatable assessment of potential ECG liabilities earlier in the discovery lead optimization process and by doing so, more rapid development of potential clinical candidates with low ECG arrhythmic risk. While effective, the advent of *in silico* tools has offered the potential for an early discovery approach that can be applied to a greater number of molecules, faster, with a reduced reliance on animals to predict non-rodent and ultimately clinical ECG liabilities.

The present study assessed a human *in silico* electrophysiological drug assay, which uses a population of human AP models, to evaluate its ability to predict the results of an anesthetized guinea pig assay currently used in pre-clinical safety assessment to assess the liability of NCEs to cause QTc interval prolongation prior to non-rodent GLP studies. The manuscripts goal was solely to predict the effects on the QTc interval which is the most common ECG parameter to be affected by various classes of molecules in early development. The strategy would be applicable to other endpoints but a greater pool of NCEs focused on conduction-related effects would be required given the lower occurrence of PR and QRS interval prolongation effects as compared to QTc interval prolongation.

The main findings of this study are:

1. *In silico* test results successfully predict the QTc interval prolongation outcome observed in the anesthetized CVGP studies. An accuracy of 85% and a specificity of 73% were obtained when PX *in vitro* ion channel inhibition data was used as inputs into the *in silico* model, compared to 75% accuracy and 77% specificity when using HTS ion channel inhibition data.
2. tPKPD modeling using *in vitro* ion channel inhibition and *in silico* output data as inputs is successful at predicting the concentration at which we observe QTc interval prolongation in the anesthetized CVGP assay. Typically, the free plasma concentration from TP compounds identified using the *in silico* model are within 5-fold of the actual CVGP QTc EC_5_ concentrations.
3. The tPKPD model results successfully re-classify many FN and positive NCEs from the *in silico* readout which increases the accuracy and specificity to 100% and 95% when using PX data and 88% and 97% when using HTS *in vitro* data, respectively.

The *in silico* drug simulations presented in this study were performed in a population of 107 human ventricular control models, built using the O'Hara-Rudy dynamic (ORd) model (O'Hara et al., 2011) as baseline and the methodology described by Britton et al. (2013) and further discussed by Muszkiewicz et al. (2016). The ORd human ventricular action potential (AP) model was chosen for this study because of: (1) the large number of human ventricular experimental data obtained from more than 140 human hearts used in its construction and evaluation; (2) its ability to reproduce and probe proarrhythmic mechanisms, including repolarization abnormalities and EMw (Passini et al., 2019), and (3) its choice within the Comprehensive *In Vitro* Proarrhythmia Assay (CiPA) initiative (Colatsky et al., 2016). It is recognized that a guinea pig specific *in silico* ventricular AP model would likely be superior at predicting the results of the CVGP. However, using a human model also gives us the ability to better predict the clinical outcome of NCEs (Passini et al., 2019) and therefore could serve two purposes. It is acknowledged that the simulated AP results may still present a challenge to predict the clinical QTc outcome given many other factors can influence it. Factors that predispose to QT prolongation and higher risk of torsades de pointes in the clinic include age, female sex, left ventricular hypertrophy, ischemia, slow heart rate, and electrolyte abnormalities including hypokalemia and hypomagnesemia (Ahnve, 1985; Khan, 2002; Locati et al., 1998; Makkar et al., 1993). Given the goal of the current study was to predict the QTc interval outcome in an early development *in vivo* model, adjustments were not made for these additional factors in the *in silico* model. The focus was rather on the main ion currents that underly the ventricular AP and how the simulated models' AP relate to the QTc interval. In addition, the specific interaction of the NCE with the ion channels such as kinetics and drug dissociation or reassociation was beyond the scope of this study. Only the drug's ion channel IC_50s_ were used as inputs to evaluate resource sparing method at this early stage of development.

Our results support that the *in silico* assay used in combination with tPKPD modeling can have qualitative and quantitative agreement with this commonly used preclinical safety pharmacology assay in early drug development. The accuracy of the *in silico* simulations to predict QTc interval prolongation in the CVGP assay is superior to the ones obtained using solely ion channel data from the PX hERG or MK-499 binding assays. In fact, the negative predictive value (NPV) when using solely PX hERG inhibition data (85%, Fig. 2A) in absence of *in silico* modeling is lower as compared to using mixed ion channel inhibition data from PX (hERG, I_Ks_, hNav1.5and hCav1.2) *in silico* (100%, Fig. 3A). This is in part due to the O'Hara Rudy (ORd) model's (O'Hara et al., 2011) increased sensitivity to hERG block (Mirams et al., 2014) which will identify ADP_90_ and/or QTc interval prolongation at lower concentrations. As such, NCEs that are identified as FN when using hERG data alone are correctly identified as TP using the *in silico* model. However, the NPV when using HTS ion channel data (MK-499 binding IC50s) is similar (80%, Fig. 3B) to the MK-499 data alone (85%, see Fig. 2B). This is likely due to lower affinity in the MK-499 binding results as compared to the PX assay results for most of the FN (compound 3, 5, 40, 57, 61 and 70). Differences in affinity between PX and HTS methods may be related to the PX assay which can reveal a functional block that is associated with potential allosteric modulation that does not displace a ligand at the ion channel pore in a standard binding assay. In addition, binding to isolated membranes may differ from intact cells (*i.e.*, access to binding site or different ionic conditions in both assays) and affinities may vary for different states of the channel.

In contrast, the positive predictive value (PPV) for PX hERG (70%), HTS MK-499 (72%), PX *In silico* (75%) and HTS *in silico* (67%) to predict QTc interval prolongation in the anesthetized CVGP assay were similar. The FP NCEs using hERG and MK-499 data alone are generally the result of not integrating mixed ion channel inhibition data from other channels (hCav1.2, hNav1.5 and I_Ks_). As such, many of these compounds are correctly classified as TN when using *in silico* simulation (NCE# 6, 11, 17, 28, 34, 35, 55, 66). The *in silico* simulation is able to distinguish between drugs which purely affect I_Kr_ and those with a multi-channel action which modify Ca^2+^ dynamics given the ability of the model to predict the effect on the EMw which has been shown to be more sensitive to drug-induced changes in Ca^2+^ transient (Passini et al., 2019). *In vivo*, the EMw is a measure of the temporal dispersion of the duration of the electrical and mechanical systole which is related to the duration of repolarization on the ECG and to the levels of intracellular Ca^2+^ in a measure of contractility. Typically, potent I_Kr_ blockers will have very little effect on simulated Ca^2+^ transient and will prolong the simulated APD_90_ and the *in vivo* QTc interval as opposed to those NCEs displaying hCav1.2 block, that do not shorten the EMw when cardiac adverse events are less likely to occur. *In vivo*, shortening of the EMw has been presented as an effective biomarker of proarrhythmia in several preclinical experimental studies (Guns et al., 2012a; Guns et al., 2012b; Morissette et al., 2016; van der Linde et al., 2010). A negative EMw, formerly referred to in the clinic as inversed QT/QS2 ratio or “QT > QS2 Syndrome”, occurs if either LV-contraction duration shortens or if the QT-interval prolongs (or a combination of both). It was shown that in the healthy myocardium, the duration of electrical systole (QT) is shorter than that of the electromechanical systole (Second heart sound; QS_2_ in the clinic), which it closely parallels throughout the range of resting heart rate (HR) (Boudoulas et al., 1982). More recently, ter Bekke et al. (2015) observed that patients with genotype-positive long QT syndrome exhibit EMw inversion and it is most pronounced in patients with documented arrhythmic events. Given the sensitivity of the EMw to detect mixed ion channel inhibition, this parameter was used to increase the sensitivity of the *in silico* model to predict QTc interval prolongation in the CVGP. However, the simulated EMw was not used, in this instance, to predict arrhythmic risk.

The remaining FP compounds identified when using the qualitative *in silico* model results (Fig. 3A and B) are a different set of NCEs from using ion channel alone (Fig. 2A and B) and are generally due to the high sensitivity of the ORd model to hERG inhibition as well as to the ability of testing high concentrations that may not be achievable and/or tolerated *in vivo*. As a result, the tPKPD model becomes useful in determining if the concentration at which we observe QTc interval prolongation is relevant. This model allows for the prediction of the concentration at which we observe meaningful QTc interval prolongation in the anesthetized CVGP assay. When the tPKPD model predicts that the concentration at which we observe QTc interval prolongation is greater than the highest concentration achieved in the CVGP with no QTc interval prolongation, the compound is correctly reclassified as TN. As a result, when the *in silico* model is used in combination with the tPKPD model, the PPV increases to 94% and 93% using PX or HTS ion channel data, respectively.

The remaining FP NCEs (NCE #20 and #59 for PX and #20 for HTS) after applying the tPKPD model are likely due to another activity that contributes to a modulation of calcium cycling which could counterbalance the effects on AP repolarization and QTc interval prolongation. For example, a mechanism that reduces intracellular Ca^2+^ such as by decreasing cAMP could counteract QTc interval prolongation and have a protective effect. As a result, it is important to understand the putative mechanism of action or potential off-target activity of NCEs to consider other mechanisms that may influence the overall repolarization outcome. Since these additional mechanisms are not standard *in silico* inputs, the model may not always accurately predict the final *in vivo* outcome. In these circumstances, it is important that NCEs continue to be tested *in vivo* prior to further progression into later development. In addition, other potential risk factors associated with QTc interval prolongation such as hypothermia (Rai et al., 2014), hypokalemia (Weiss et al., 2017), glucose levels/hypoglycemia (Sertbas et al., 2017), metabolites active on hERG, accumulation of test article in the myocardium, and/or inhibition of the translocation/trafficking of the hERG channel to the membrane (Eckhardt et al., 2005) will not be evaluated *in silico* unless this is known *a priori* and is integrated into the model. Moreover, additional cardiovascular information provided from an *in vivo* assay such as hemodynamic assessment, changes in cardiac contractility, effect on autonomic nervous system (ortho- & parasympathetic) balance and/or cardio-renal baroreflexes, and effects on cardiac conduction are not currently predicted from single cell *in silico* assays, as the ones considered in this study. However, novel frameworks to run multiscale simulations are currently being developed (Lopez-Perez et al., 2019; Vigmond et al., 2009), also including computer model of human contraction (Fritz et al., 2014; Land et al., 2017), which will allow to compute additional *in silico* biomarkers.

The anesthetized CVGP assay has previously been compared to results in the non-rodent (dog and monkeys) and the clinic (QTc) to assess its usefulness in early derisking (Morissette et al., 2013). High levels of sensitivity (75–100%), and specificity (100%) was observed. The overall accuracy of the anesthetized CVGP assay was very high (92–100%), given the outcome of the non-rodent telemetry models were well predicted by the CVGP. In addition, there was a robust correlation between the CVGP and non-rodent free plasma concentrations (≤ 10-fold difference) that produced comparable changes in QTc interval in non-rodent and the clinic. Consequently, it could be argued that *in silico* results in combination with tPKPD modeling could also be used to predict meaningful free plasma concentrations that would prolong QTc intervals in the non-rodent GLP assays and FIH clinical studies.

## 5 Conclusions

This study demonstrated that the combination of an *in silico* cardiac electrophysiological model and a tPKPD model is highly predictive, sensitive (low rate of FN NCEs), and specific (low rate of FP NCEs) for predicting QTc interval prolongation in a commonly used *in vivo* model, the anesthetized CVGP. Simulated APD_90_ and EMw changes observed *in silico* are qualitatively predictive of QTc interval changes in the anesthetized CVGP. Using tPKPD modeling to predict threshold QTc prolongation concentrations in the CVGP correctly re-classified NCE's and increased the overall positive predictive value (PPV). Ultimately, these comparative approaches demonstrated the predictive value of an *in silico,* tPKPD model to determine *in vivo* QTc prolongation. Consequently, we should consider the use of such approaches early in the NCE discovery research process to increase throughput, query a broader range of compounds to better assess QTc risk liabilities in an early *in vivo* derisking model, and potentially reduce animal, costs & resource intensive *in vivo* studies. Finally, it would be consistent with the “3Rs” paradigm for animal research and should enable a faster advancement of NCEs into preclinical and clinical development.

## Declaration of Competing Interest

The authors declare no conflicts of interest.

## Appendix A Supplementary data

Supplementary material Image 1
