# Supplementary material for: Combining an in silico proarrhythmic risk assay with a tPKPD model to predict QTc interval prolongation in the anesthetized guinea pig assay
Source: Toxicol Appl Pharmacol. 2020 Mar 1;390:114883. doi: 10.1016/j.taap.2020.114883 (PMC7322544; doi:10.1016/j.taap.2020.114883)
Supplement: Supplementary file 1 — Supplementary material [file mmc1.docx]

**Supplemental Material**

**Workflow: Determining the predictive capacity of an in silico model alone or an in silico model in combination with a tPKPD model to predict the CVGP QTc interval prolongation results.**

**STEP 1**

Generate IC_50_’s using either PatchXpress® (PX) Electrophysiology and/or High throughput screening (HTS) methods for a compound. IC_50s_ for all compounds are found in table 3.

| Approach  Parameter (IC_50_ µM) | PX | HTS |
| --- | --- | --- |
| High Throughput MK-499 Binding |  | × |
| High Throughput hNav1.5 |  | × |
| High Throughput ICa* | x | × |
| PatchXpress INa (hNav1.5) | × |  |
| PatchXpress I_Kr_ (hERG) | × |  |
| PatchXpress I_Ks_ (KCNQ1/ KCNE1) | × |  |

Ion channel data generated by either PX or HTS methods

*ICa data generated with HTS method is used in both approaches (PX and HTS)

Example: Inhibition data generated for various cardiac currents (PX hNav1.5, PX hERG, PX I_Ks_ and HTS Cav1.2) by moxifloxacin (Compound #7 in tables 3, 4 and 5 of manuscript)

**STEP 2**

Enter ion channel data generated in STEP 1 (IC_50s_) into the in silico model and generate the effects on simulated APD_90_, CTD_90_ and EMw over a range of concentrations.

Example: Effects of varying moxifloxacin (Compound #7 in tables 3, 4 and 5 of manuscript) concentrations on the A) simulated membrane potential and B) intracellular calcium signals. APD_90_, CTD_90_ and EMw moxifloxacin concentration response is illustrated in (C).


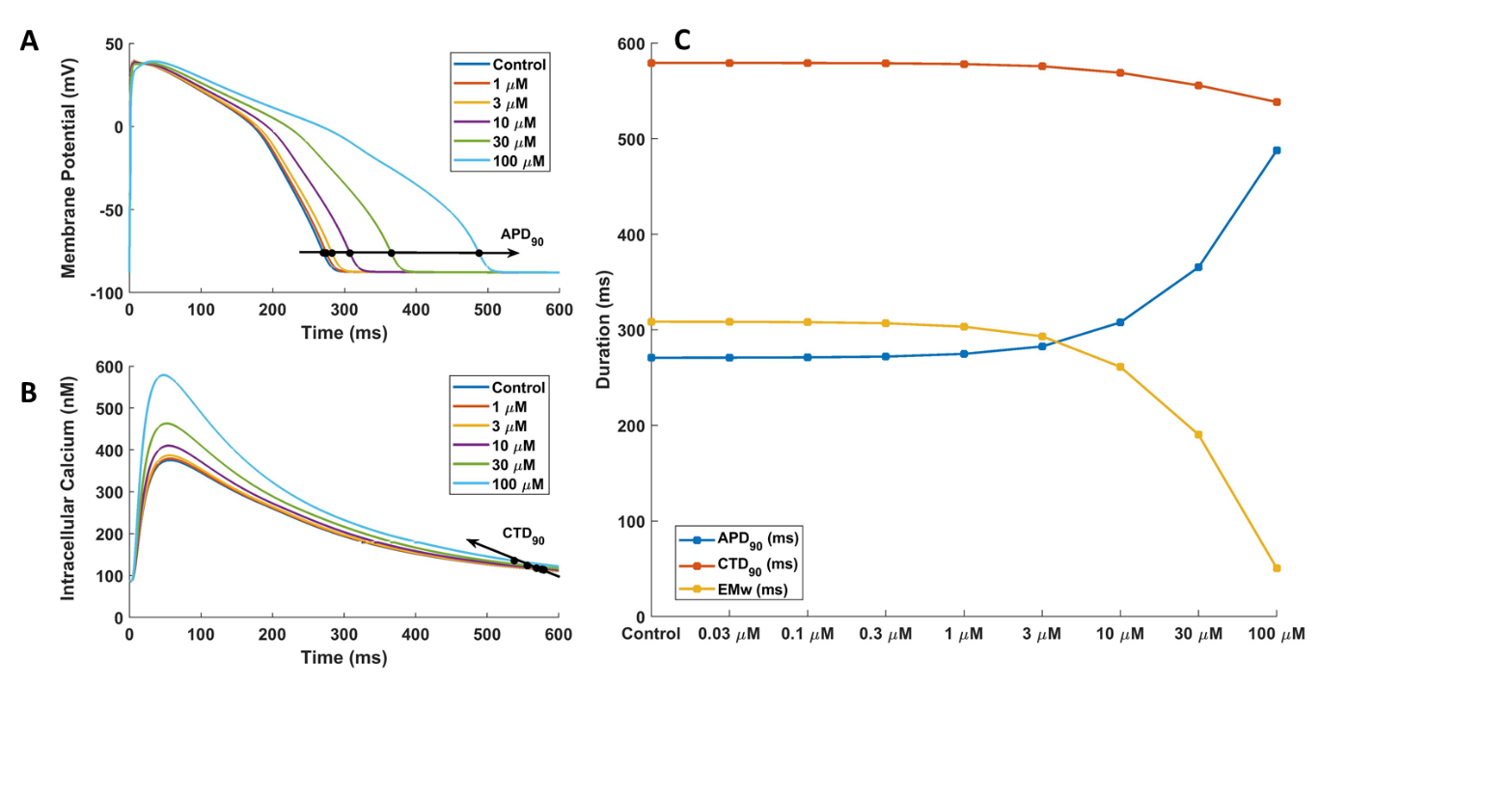


**STEP 3**

Determine the predictive capacity of an in silico model alone to prolong the QTc interval in the CVGP. This is accomplished by comparing the qualitative directional effects from the simulated APD_90_ and EMw generated in Step 2 to the effects on the directional effects on the QTc interval in the CVGP. Directional effects on simulated APD_90_, EMw and CVGP QTc are found in table 5 for all NCEs. Matrix results are summarized in Figure 3 A (PX) and B (HTS) of the manuscript.

**Example of matrix result determination using only in silico modeling**

| **Matrix Result** | **In Silico** | | **CVGP** |
| --- | --- | --- | --- |
|  | **Action Potential** | **EMw** | **QTc interval** |
| True Positive | ↑ | ↓ | ↑ |
| True Negative | No effect | No effect | No effect |
|  | ↓ | ↑ | No effect |
| False Positive | ↑ | ↓ | No effect |
| False Negative | No effect | No effect | ↑ |
|  | ↓ | ↑ | ↑ |

↑: ≥5% increase in APD90 or EMw

↓: ≥-5% decrease in APD90 or EMw

**STEP 4**

Selected parameters from Table 2 (in vitro ion channel IC_50s_ and In silico outputs parameters from the simulated action potentials) are entered into the tPKPD model for all true positives and false positive NCEs. The tPKPD model will generate/predict the free plasma concentration at which there is a 5% increase in QTc interval in the CVGP (EC_5_). tPKPD results for all NCEs are found in table 5.

**STEP 5**

Re-evaluate the predictive capacity of the in silico model results in combination with the tPKPD model to prolong the QTc interval in the CVGP. Compare the QTc EC_5_ obtained from the CVGP and reclassify compounds (TP, TN, FP, FN), if needed, using the projected EC_5_ results from the tPKPD model for matrix comparison. tPKPD and CVGP QTc EC_5’s_ are found in table 5 for all NCEs. Matrix results are summarized in Figure 3 C (PX) and D (HTS).

| **Matrix Result** | **In Silico** | | **CVGP** | **tPKPD EC_5_ result** |
| --- | --- | --- | --- | --- |
|  | **Action Potential** | **EMw** | **QTc interval** |  |
| True Positive | ↑ | ↓ | ↑ | Predicted tPKPD EC_5_ is within 5-fold of the actual CVGP QTc EC_5_ |
| True Negative | No effect | No effect | No effect | For FP compounds: If predicted tPKPD QTc EC_5_ concentration is greater than the free plasma concentration achieved in the CVGP (with no QTc interval effect), the compound is re classified as true negative |
|  | ↓ | ↑ | No effect |  |
| False Positive | ↑ | ↓ | No effect | If predicted tPKPD QTc EC_5_ concentration is lower than the highest concentrations achieved in the CVGP which did not increase the QTc interval, the compound is considered FP. |
| False Negative | No effect | No effect | ↑ | No tPKPD adjustments are made for compounds considered negative using the qualitative in silico data alone. |
|  | ↓ | ↑ | ↑ |  |

↑: ≥5% increase in APD90 or EMw

↓: ≥-5% decrease in APD90 or EMw

**Example:**

CVGP moxifloxacin (Compound #7 in tables 3, 4 and 5) results: Panel A presents the percent change from baseline in QTcVdW over the course of the i.v. drug infusion (60 min) for vehicle or [moxifloxacin](https://www.sciencedirect.com/topics/pharmacology-toxicology-and-pharmaceutical-science/moxifloxacin) at 3, 7 and 20 mg/kg (infusion 1, 2 and 3, respectively). Panel B represents the PK/PD effects of moxifloxacin on QTc.

**Panel B**

**Panel A**


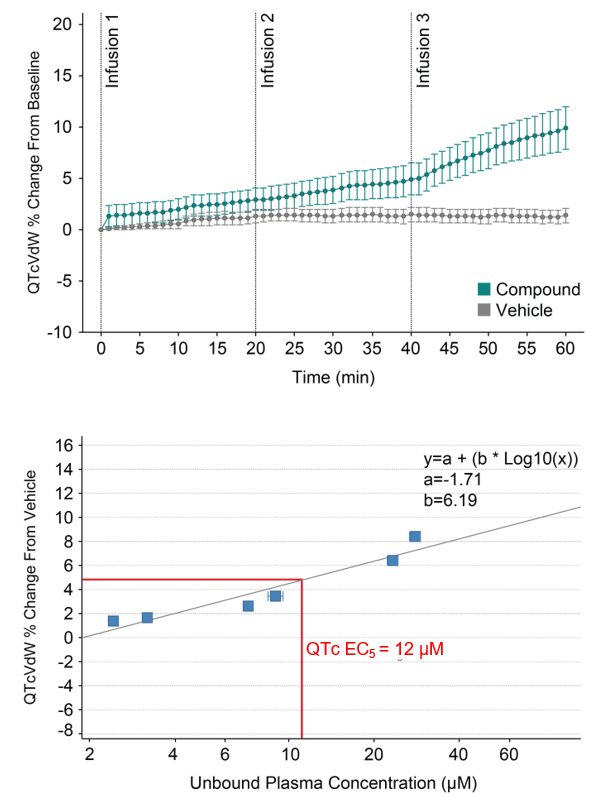


In summary, moxifloxacin is considered a true positive to prolong the QTc interval in the CVGP. Its Simulated APD_90_ increases, simulated EMw decreases and the tPKPD model predicts a QTc EC_5_ that is within 5-fold of the actual CVGP EC_5_.

Summary of moxifloxacin results:

| **Compound** | **CVGP QTc EC_5_ (µM)^1^** | ***In Silico* APD_90_ directional effect** | ***In Silico* EMw directional effect** | **tPKPD QTc EC_5_**  **(µM)^1^** | **Confusion matrix result adjusted with tPKPD results** |
| --- | --- | --- | --- | --- | --- |
| **7 (Moxifloxacin)** | **15** | **↑** | **↓** | **12** | **TP** |

^1^The listed concentrations are free plasma concentrations
